# Characterizing the spatial structures of competing football teams

**DOI:** 10.1038/s41598-025-97765-y

**Published:** 2025-10-09

**Authors:** Guy Amichay, Hugo Silva, João Brito, Rui Marcelino

**Affiliations:** 1 Department of Engineering Sciences and Applied Mathematics, Northwestern University, Evanston, IL USA; 2 Northwestern Institute on Complex Systems, Northwestern University, Evanston, IL USA; 3 NSF-Simons National Institute for Theory and Mathematics in Biology, Chicago, IL USA; 4 Research Center in Sports Sciences, Health Sciences and Human Development, Elite Research Community, CIDESD, Maia, Portugal; 5 University of Maia, Maia, Portugal; 6 FPF Academy, Portuguese Football Federation, Oeiras, Portugal; 7 CIPER, Faculdade de Motricidade Humana, Universidade de Lisboa, Lisboa, Portugal

**Keywords:** Football analytics, Player trajectories, Collective movement, Team shape, Convex layers, Human behaviour, Physiology

## Abstract

Progress in football analytics is hampered by two main reasons: the unavailability of detailed datasets; and a dearth of meaningful metrics that would aid in gaining insight into the collective action of teams. We analyzed a dataset consisting of the full trajectories of all players, from twelve complete matches from three major international tournaments. We studied the teams’ spatial structure, a topic that has been, to date, relatively unexplored. What has typically been analyzed is the teams’ convex hull which gives a measure for the overall spread of the team in space, but which ignores the contributions of the players that are within it. Here we computed, for the first time, also the convex hull of those interior players. We then calculated the ratio of the area of the inner convex hull over the area of the outer convex hull, which gives us a single number that encapsulates the geometry of a team for a given point in time. We find a relatively general pattern—the ratio of the areas of these two convex layers almost always dies out at ~ 0.5 (i.e., there are almost no cases where the area of the inner layer exceeds 50% of the area of the outer layer). This result proves to be robust: for instance, it appears in both attacking and defending layouts (which are typically associated with very different team spatial structures). While it remains unclear whether a layer ratio value outside the observed range has practical implications, our findings provide a foundation for future research into the spatial geometry of team sports and its potential strategic relevance.

**Supplementary Information:**

The online version contains supplementary material available at 10.1038/s41598-025-97765-y.

## Introduction

In recent years, advancements in technology and data-driven methodologies have revolutionized sports analytics^1–3^. The utilization of positional data, commonly known as player tracking data, has become essential in this field^4^ by improving our understanding of team dynamics, player strategies, and overall performance. For example, choosing a playing formation with three defenders may elicit higher physical demands compared with a formation with four defenders^5^. Moreover, practitioners and researchers can evaluate technical performances by analyzing the success of shots from specific positions or determining how many opponents a pass outplays based on longitudinal coordinates^6^. Furthermore, data analysis can go even further, where tracking players’ movements on the field helps to understand the complexities of tactical play and strategic decision-making^7–9^. In fact, player tracking data provides a comprehensive map of players’ spatial interactions during matches, offering insights that surpass those derived from traditional notational analysis methods. This sophisticated analytical approach allows for an in-depth understanding of team coordination and tactics in both offensive and defensive contexts^1^.

Beyond football, spatial analytics have been extensively explored in other team sports. For instance, in Australian football, studies have utilized spatial analysis methods like the ‘Talent Tracker’ to map regional talent production, revealing spatial patterns in player development^10^. Similarly, metrics such as convex hull area have been used to examine team spread and the evolution of player roles during gameplay^11^. In American football, predictive models derived from spatial features, such as player distances to anchor objects like the ball, have enhanced game outcome forecasts^12^.

While these examples demonstrate the versatility of spatial analytics across sports, the focus now turns to football, where the implications of player positioning, ball distribution, and movement patterns are particularly profound^13–15^. In football, reduced player dispersion is often linked to short passing strategies, whereas greater dispersion correlates with an increased use of long passes^16^. Defensively, teams contract their formations to limit the opposition’s space while maintaining synchronization, a critical factor when facing high-quality opponents with collaborative strategies^4^. This synchronization becomes even more critical when facing high-quality opponents who employ a collaborative approach^17^.

Therefore, if a player decides to perform a movement that disrupts the space intra-team, a teammate may be exposed to high demands to compensate for that movement^18^. Despite these innovative analyses, the challenge of translating new knowledge into practical applications remains significant^19^. One specific issue is that, although interesting, some analyses yield complex mathematical results that are difficult for practitioners to implement and interpret. We are thus looking for simple metrics that are easy to understand.

To date, a common measure of the spatial distribution of a team is to compute its convex hull. Imagine a very big rubber band, wrapped around the team, with the players holding it with their bodies. If the team spreads out, the total area of the rubber band (the convex hull) grows; conversely if they come together to form a more dense clump, it shrinks. The problem with such a view though, is that typically there are players within the convex hull that aren’t contributing to it (the rubber band isn’t in contact with them). Here we take one step further, and compute the convex hull of those inner players (so-called “convex layers”). We then simply look at the ratio of these areas, which encapsulates the whole (or almost the whole) instantaneous spatial structure of a team (the positions of all the players of the team, for a given moment) to one interpretable number—putting forth a new metric for football analytics. Note that this is not intended to replace the usage of the external convex hull itself; this could accompany the convex hull measure, as they each communicate different information. While traditional spatial metrics like the convex hull have provided valuable insights into overall team dispersion, they often fail to account for the intricate contributions of interior players to the team’s spatial structure. Interior players are pivotal in maintaining cohesion, controlling transitions, and sustaining tactical balance. Addressing this gap, our approach introduces a novel layer-based perspective to complement existing metrics, offering a more granular view of team dynamics. By focusing on these interior layers and their relationship to the overall team structure, we aim to provide new opportunities for actionable tactical insights that are both theoretically robust and practically relevant for advancing spatial analytics in football.

## Methods

### Dataset

This study is data-driven—we analyzed a novel and unique dataset that allows us to reach conclusions that wouldn’t be available otherwise. The dataset consists of positional data, including player and ball coordinates (x and y) obtained through optical tracking provided by the official supplier of the competition (data was provided by Tracab). Overall, we analyzed twelve national team matches of one team against different opponents across three international competitions. Specifically, the sample encompasses matches from UEFA Euro 2016 (*N* = 7), the 2018 FIFA World Cup (*N* = 2), and UEFA Euro 2020 (*N* = 3). This includes both group stage matches (*N* = 8)—which offer insights into early-stage tactical approaches—and matches from the knockout stages (*N* = 4), providing a holistic perspective on the team’s behavior throughout various phases of these prestigious competitions. The selected sample represents a balance between breadth and depth, capturing a diverse set of opponents, tactical contexts, and performance pressures. These matches were chosen to encompass a variety of competition formats and stages, enabling a robust examination of the team’s tactical adaptability and performance under varying levels of competitive intensity. Moreover, focusing on international competitions ensures a high level of consistency in data collection, as these tournaments involve standardized conditions, such as venue quality and officiating, which minimizes external variability that might otherwise confound the analysis.

**Fig. 1:**
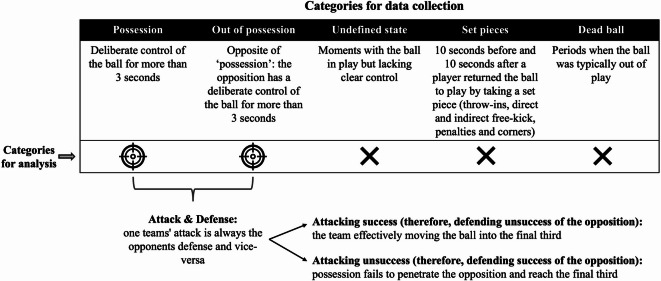
Diagram of how the data was categorized and analyzed.

The data include also players that came on as substitutes and replaced other players. In each match, we analyzed the whole match (two halves of 45 min including stoppage time). We did not include extra time in case that occurred. In addition to the positional data, a team of analysts observed all matches to identify distinct phases of the game, categorized based on ball status: deliberate control of the ball for more than 3 s (possession); moments with the ball in play but lacking clear control (undefined state); 10 s before and after set pieces (during set pieces); and periods when the ball was typically out of play (dead-ball situations) (Fig. 1). This information was then synchronized with the positional data and, for this study, only the positional data of the moments in possession was considered.

### Data analysis

**Fig. 2:**
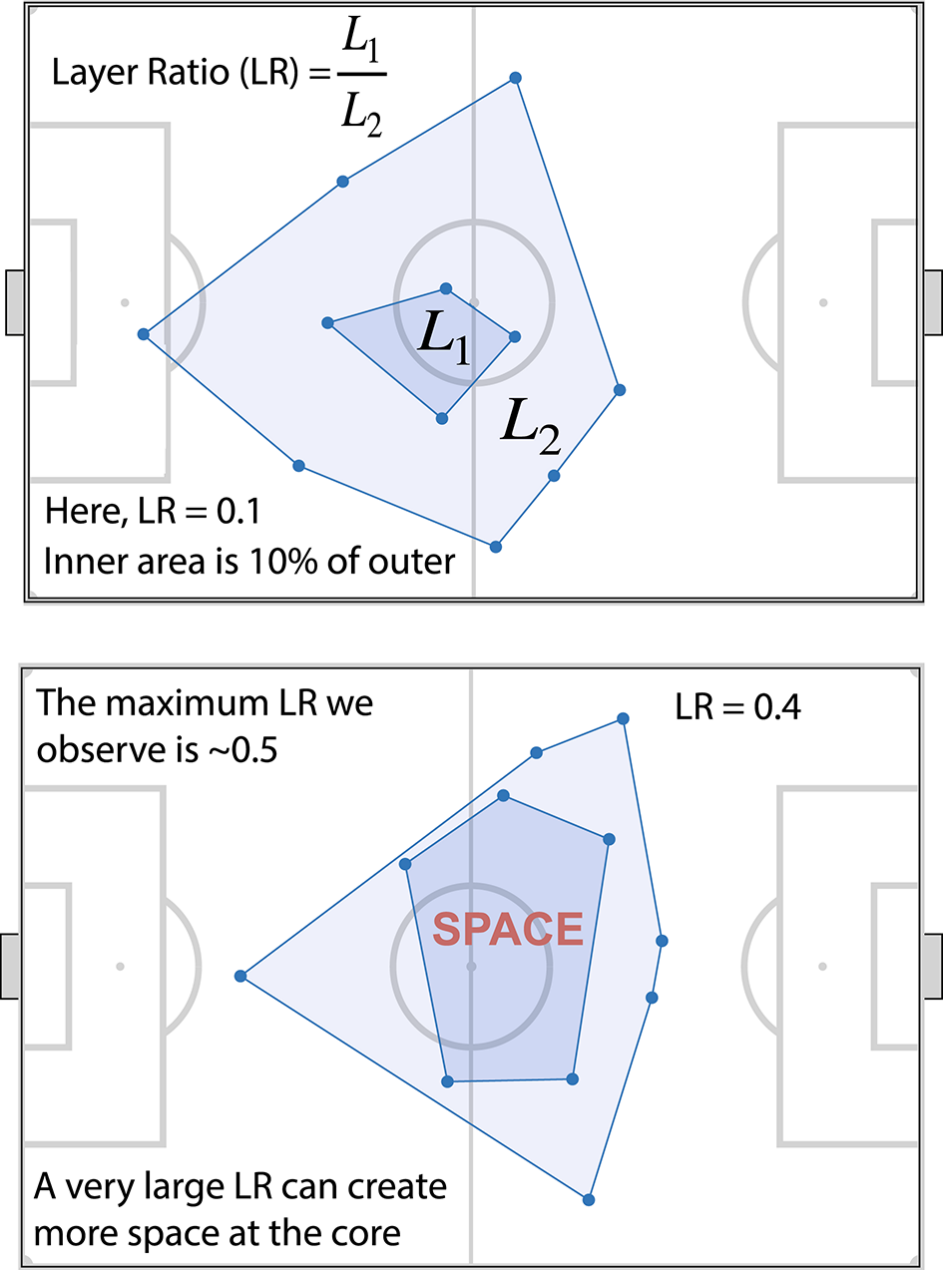
Overview of the analysis methodology. In both plots we overlay the data on a schematic of a football pitch. Top plot shows the convex layers of one team (all 11 players; including the goalkeeper) from a random moment (frame) in a match. Here we show the definition of the layer ratio (LR), the ratio of the areas of the two layers. In the bottom plot we show an example of one of the larger LR we observed in our data.

Data was analyzed using Matlab version 2020a. To calculate the layer ratio of a team for each point in time, we performed the following steps (for each frame separately):

1. Computed the teams’ outer convex hull (we specifically used the built-in convhull function in Matlab). By doing this we essentially computed the instantaneous 2D convex hull from the 2D positions of the players of that team. We did this for all 10 outfield players (or all 11 when we included the goalkeeper).
2. We repeated this step for the inner layer (computing an additional convex hull), only doing so with the remaining players (*those that lie within the outer convex hull* and weren’t contributing to it; Fig. 2, and see SI Appendix S3 for additional examples).
3. Each of these convex hull’s has an associated area (the portion that they occupy on the pitch); the layer ratio is the ratio of these areas—we divided the inner area by the outer area (See Fig. 2, top plot). It is a dimensionless quantity, technically ranging from 0 (if there were zero to one players contributing to the inner layer) to 1 (if the inner layer would fit perfectly into the outer layer—which is practically impossible).

We analyzed windows of time that were in- and out-of-possession (that is, excluding transitions, set pieces and dead-ball situations). One teams’ attack is always the opponents defense and vice versa. But we took an additional step. Beyond splitting the data up according to different modes of play, we also wanted to separate it according to some ball-progressing measure. That is, when a team has possession, are they successfully progressing the ball toward their opponents’ goal? Conversely, is the team out of possession successful at preventing them doing so? More precisely, we split the pitch into thirds (the defensive area, the middle of the pitch, and the final third). A successful ball-progression was defined when a team managed to progress the ball from the 1st or 2nd third into the final third. We determine this by checking where the possession started and where it ended. Accordingly, if they didn’t manage to do so, that would count as an unsuccessful ball-progression attempt. The defending team in these cases is the opposite: if the attacking team failed, they succeeded and vice versa. We acknowledge that this is a crude distinction that won’t always be true (e.g., sometimes defending teams choose a tactic where they “invite” the opponent to progress the ball into the final third) but still it should be a reasonable rough approximation.

We eventually pooled data from multiple different occasions (i.e., different attacking and defending attempts) and looked at the resultant distributions. We split the game up in a relatively conservative approach, establishing a minimum duration of 3 s for defining a defending or attacking phase. This allowed us to exclude match situations where possession isn’t clearly defined. (Our approach can also be applied to specific moments, such as identifying the LR when the team gains or loses possession.) We sampled once every 1 s (one frame every 25 frames). We computed a kernel density estimate (KDE) of these distributions, with which we derived probability density functions (PDF)—essentially normalized distributions (the area under the curve equals 1), allowing us to compare distributions that were drawn from different sized datasets.

## Results

To elucidate the internal structure of the team (relative to its’ “external” players) we looked at the convex layers of each team. In other words, we can compute not only the convex hull around the perimeter of the whole team, but also a convex hull for the remaining (inner) players (a point if it is one player; a line if it is two; triangle for three and so on). Note that technically, there could be also a third layer. As usually this won’t be the case due to the relatively small number of players on a football team, we start by computing only the two main layers and relax this assumption later on.

This method, to the best of our knowledge, has not been applied in football or other team sport analysis beforehand. Now we can ask: how much of the total area of the outer convex hull is actually occupied by the inner one? This procedure yields a single number (the ratio of the inner and outer areas, the layer ratio—LR) that reduces the entire instantaneous geometry (or at least almost the entire geometry) of a team at that point in time.

**Fig. 3:**
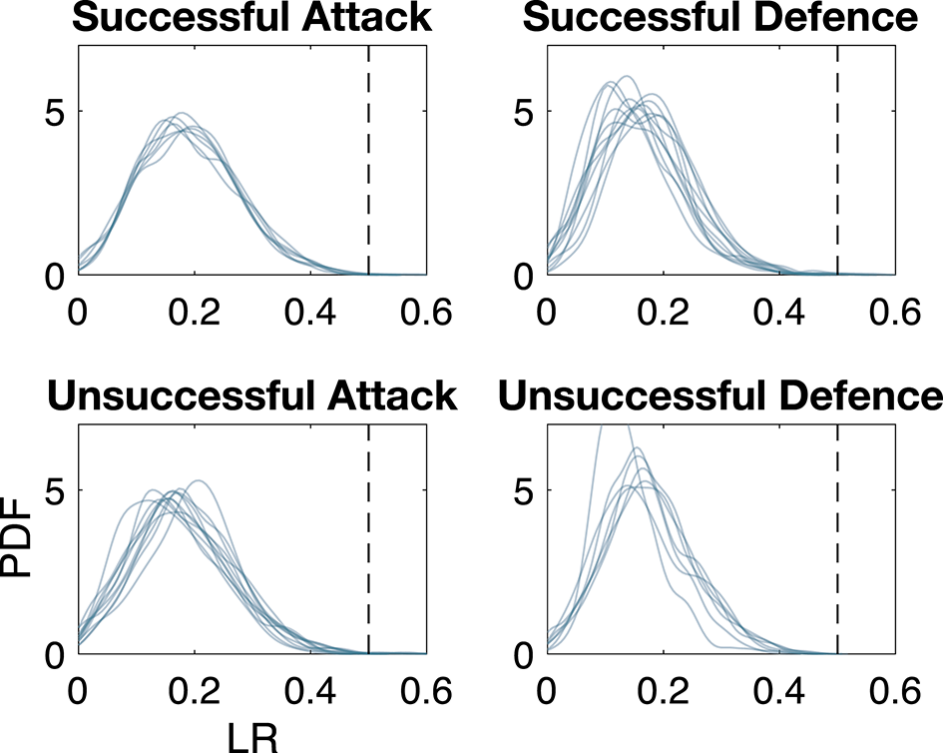
Ratios of areas of the convex layers of all players, including the goalkeeper. Distributions (probability distribution functions obtained from kernel density estimates) of the ratio of the area of the inner convex hull over the area of the outer—each curve corresponds to data from specific events from one match. A few cases were discarded for having an insufficient amount of data (< 500 data points). Dashed vertical lines show approximately where all the distributions end at 0.5.

When looking at the overall distributions of the LR we reveal a relatively universal pattern; regardless of whether teams are in attack or defense (which typically result in drastically different spatial structures: usually in defense teams are more dense, and in attack teams are more spread out in space^8^) or even whether these were successful ball-progression attempts or not, the ratios peak at ~ 0.18 (i.e., the inner area is 18% of the outer one), and these distributions always die out at ~ 0.5. In other words, we almost never observed cases where the ratio is > 0.5 (Fig. 3). The typical number of players that created the inner layer were 3 (~ 12.5%), 4 (~ 51%) or 5 (~ 31.5%) players, and for the outer layer 5 (~ 24.5%), 6 (~ 41.5%) or 7 (~ 24.5%) players.

Note that we conducted this calculation considering all 11 players (i.e., including the goalkeeper). This approach was adopted because modern goalkeepers increasingly participate in the build-up of play and often contribute to the team’s spatial organization during possession phases. However, when teams transition into offensive play, the goalkeeper frequently remains near the goal, potentially reducing their influence on the spatial dispersion of the team. Hence when considering the goalkeepers in this analysis, the outer convex hull would likely be skewed. To probe the robustness of our results, we conducted the same calculation also excluding the goalkeepers (i.e., only the 10 outfield players; SI Appendix S1). We find, maybe a bit counterintuitively, that the distributions are shifted to the left, likely due to the fact that the players that contribute to the different layers might also be swapped. Nevertheless, the cutoff of the distributions (where they die out) actually appears similar to what we observed with the goalkeepers—highlighting that these findings aren’t sensitive to this choice. These parallel analyses allow researchers to select the approach that best suits their tactical context, emphasizing different phases of possession. At this stage, we do not advocate a definitive preference; instead, we provide both options to support future investigations.

We also went one step further: could there be a third layer? We typically find 0 to 2 players within the inner layer (we refer to these as “core players”). But does the LR depend on this number? We computed separate LR distributions according to the number of core players (SI Appendix S2) and found that the median value of LR in cases of no core players (zero players at the core) was always lower when compared with cases with one core player, in all our matches (there are no conclusive results when comparing one vs. two core players, but there are fewer data in these cases). Finally, we also compared the LR according to the position of the ball (if it was at the focal teams’ own half or the opposition’s half) and once again saw no clear differences (SI Appendix S4).

## Discussion

In this study we investigated the spatial structure of football teams during high-profile matches by introducing a novel ratio, the layer ratio (LR). This ratio quantifies the relationship between an outer convex layer, defined by connecting the outermost locations of the 11 or 10 players (with or without the goalkeeper), and an inner convex layer (defined by connecting the points of the remaining players). In general, the higher the LR ratio, the closer the players in the inner layer are to the outer layer.

We also computed the LR under different game scenarios, employing a conservative approach with a minimum duration of 3 s for defining a defending or attacking phase. This decision ensured the exclusion of phases where possession was not clearly defined, such as during transitions immediately after possession changes. By doing so, we increased the robustness of our findings, focusing on clearly defined tactical phases. This methodological choice strengthens the reliability of the observed patterns and provides a framework adaptable to analyzing specific moments, such as possession gains or losses.

We found what appears to be a robust universal pattern—the area of the inner layer (the convex hull of the central players) is always up to ~ 50% of the outer layer. We “perturbed” our system with a number of variations; our results suggest that the LR always dies out at ~ 0.5, regardless of: (i) the phase of play (attack or defense); (ii) whether the ball-progression attempt was successful or not; (iii) whether the goalkeepers were taken into account or not; and (iv) the stage of the competition (group stage or knockout stage). This evidence suggests that this finding is robust and may be found in other circumstances.

A previous study by Forcher et al. (2024)^20^ examined player proximity during the defensive process, finding that the distance between the defensive and midfield lines was shorter (10.22 ± 3.76 m) than between the midfield and attacking lines (13.30 ± 4.22 m). However, these distances can vary depending on team strategy. For instance, a high-pressing approach may reduce inter-line distances, while deep defending can also compress these spaces. Our current study introduces a novel approach that facilitates the rapid assessment of whether teams are occupying significant portions of the peripheral zone, thereby creating a narrow corridor between inner and outer players. If such a configuration is observed, it may indicate a vulnerability in the team’s defensive positioning, potentially exposing them to exploitation by the opponent. On the other hand, is this necessarily always true, or could teams adapt to new forms of collective defending? Also, are such gaps necessarily an issue for attack?

A recent review by Low et al. (2020)^21^ has highlighted the need for interpretable measures of the multitudes of intricate interactions co-occurring during football matches. They mention a plethora of approaches (e.g., “effective playing space”^22^, stretch index^23^ and many more). Moreover, the growing popularity of deep learning approaches has led to a rise in various new metrics, such as the “expected goals” metric^24^. However, the authors also note the difficulty of deriving meaningful insights from these metrics, highlighting the ongoing need for metrics that are both meaningful and interpretable. In contrast, our research offers a practical advantage due to its simplicity, owing to the fact that we propose a single ratio ranging from zero to one. This straightforward metric offers a transparent and comprehensible indicator of the geometry of the team as a whole, while also allowing ease of implementation, in both analytical and coaching settings.

We reflect on how our findings could be readily adopted in actual play. It is important to consider that allowing a team to have large open spaces (a large core) is not necessarily detrimental and warrants further exploration. That said, we often see that there are a few players actually occupying that space (1–2 players)—but we very rarely saw cases of 3 core players. Again, is this fundamentally an issue? Football formations are rapidly changing. It is easy to imagine formations where we observe 3 (or even more) core players, essentially forming a 3rd layer. This will allow the LR (as we’ve defined it, between the 1st and 2nd layers) to easily break the “ceiling” we’ve observed here of 0.5.

Although we observed a relatively clear result here, we should be cautious to interpret this as a universal truth. The total number of games analyzed, or the number of games during the group vs. knockout stages, is not particularly high. Future studies can assess if these ratio values are replicated in other contexts. For instance, it would be interesting to test this also with matches from competitive clubs. If we still see a similar cutoff, that would further suggest that our results are quite general. If results appear different—that could be a signature for the different playing styles of clubs vs. national teams.

Our method may also be adapted and tested in other team sports, acknowledging their distinct spatial dynamics. For example, in basketball, Zuccolotto et al. (2021)^25^, demonstrated how spatial statistical methods, such as court partitioning, can inform shooting strategies by characterizing scoring probabilities in different court zones. In hockey, Bartseva et al.^26^ highlighted the complex relationship between intensive training, spatial ability, and cognitive performance, emphasizing how sport-specific demands influence spatial reasoning. Similarly, rugby has been shown to benefit from advanced analytical tools, as Passos et al. (2006)^27^ employed artificial neural networks to reconstruct 3D performance spaces and identify key patterns in game dynamics. These examples illustrate the potential for broader applications of spatial metrics across sports. However, practitioners and researchers should prioritize utilizing the LR ratio calculation procedures while adapting to the specific constraints and dynamics of each sport. For instance, basketball and hockey, which involve fewer players and faster transitions, may not be applicable, as typically there wouldn’t be an inner layer. Rugby and American football, on the other hand, have larger teams. But with their distinct phases and role-specific spatial structures, will likely require context-sensitive adaptations.

As a final note, this work is just a first step in elucidating the dynamics of team structure. By computing the LR distributions, we observed a very robust and clear pattern—the fact that they don’t exceed 0.5. Distributions carry much more information though, and future studies could examine them in more detail. In addition, we can think of many new avenues utilizing the LR approach: how does one teams instantaneous LR depend on the opponents? Where in the pitch (and in what situations) do we typically see smaller LR and when larger? How does the location of the inner convex hull relative to the outer convex hull affect team performance? These are just a few of the directions that this type of analysis could take. As such, we hope this work both motivates new studies, as well as stimulates football practitioners (coaching and managerial staff) to consider what we view as untapped potential in the game.

## Electronic supplementary material

Below is the link to the electronic supplementary material.

Supplementary Material 1

## Data Availability

The datasets used and analyzed during the current study are available from the corresponding author (Rui Marcelino-rui.marcelino@fpf.pt) on reasonable request.
