# Supplementary material for: Characterizing the spatial structures of competing football teams
Source: Sci Rep. 2025 Oct 9;15:35217. doi: 10.1038/s41598-025-97765-y (PMC12511544; doi:10.1038/s41598-025-97765-y)
Supplement: Supplementary file 1 — Supplementary Material 1 [file 41598_2025_97765_MOESM1_ESM.docx]

**Supplementary information for “Characterizing the spatial structures of competing football teams”**

**S1: Ratios of areas of the convex layers of all outfield players (excluding the goalkeeper).** Distributions (probability distribution functions; obtained from kernel density estimates) of the ratio of the area of the inner convex hull over the area of the outer--each curve corresponds to data from specific events from one match. A few cases were discarded for having an insufficient amount of data (<500 data points). Dashed vertical lines show approximately where all the distributions end at 0.5.

**
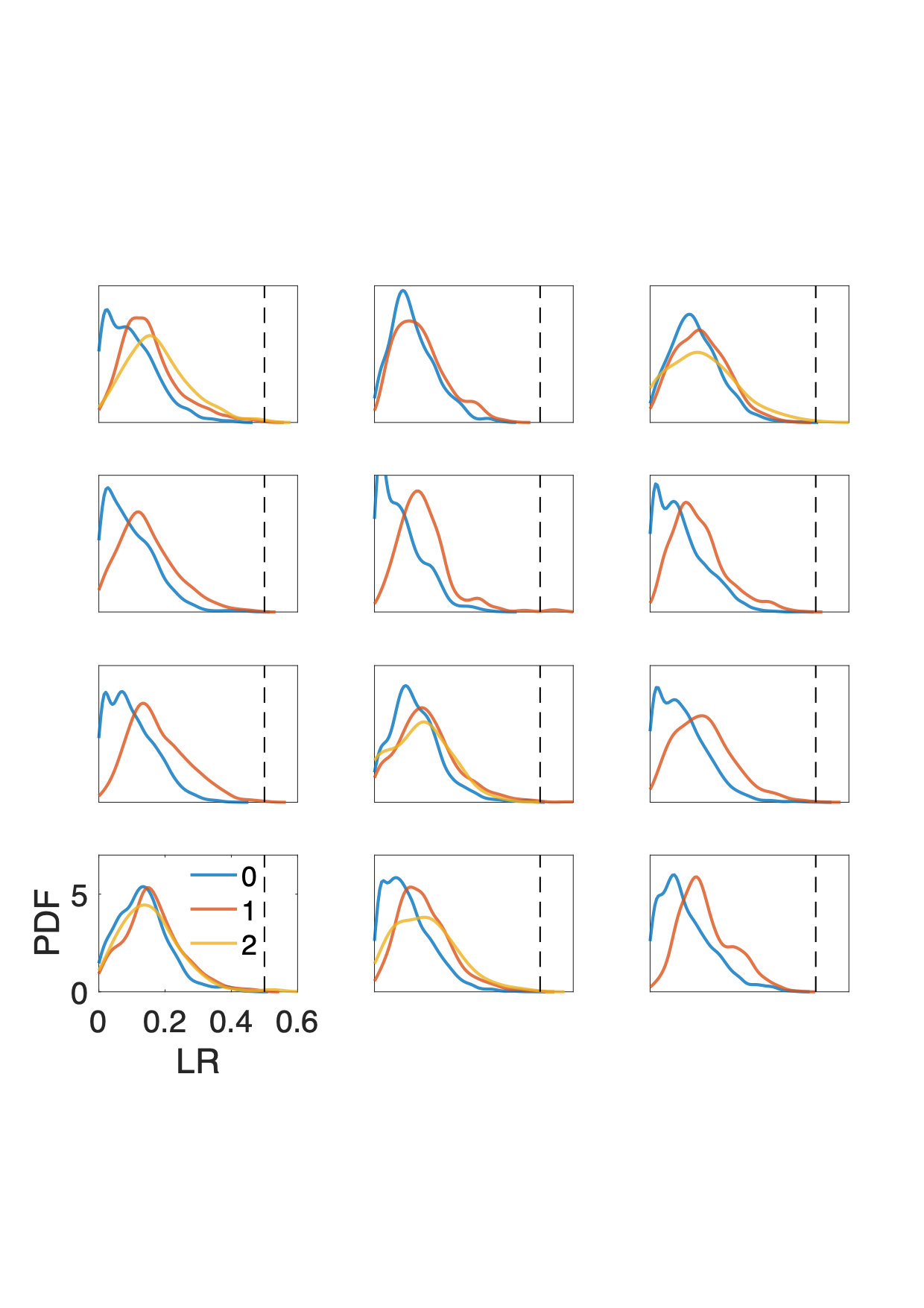
**

**S2: LR distributions separated according to the number of players within the inner layer.** Here we used only the 10 outfield players. Blue curves are for cases when there weren't any players within the inner layer; red curves are for cases with one player; and yellow curves are for cases with two. Each plot corresponds to one match. Some cases were discarded for having an insufficient amount of data (<100 data points; here we used a lower threshold to allow us to observe a few examples of 2 core players). Dashed vertical lines show approximately where all the distributions end at 0.5.


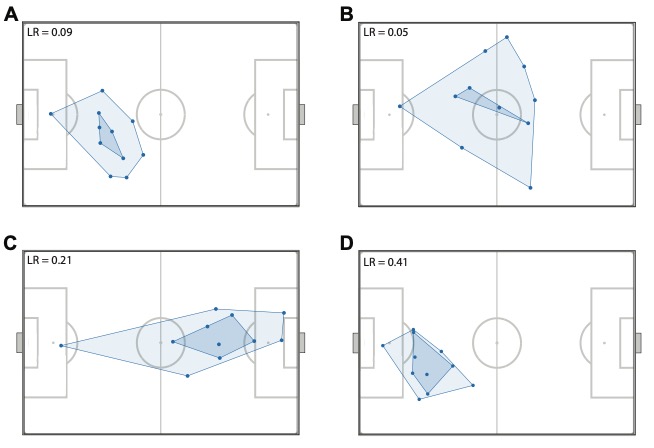


**S3: LR examples.** (A and C) LR displayed for a moment where the team was in a more defensive formation (with all the players within their own half, A), and below it for comparison an example of an offensive formation (with all the players except the goalkeeper within the opponents’ half, C). In both cases the team was attacking from left to right. (B and D) examples of very low and high LR. An LR of ~0.05 (B) vs an LR of ~0.41 below it for comparison (D).


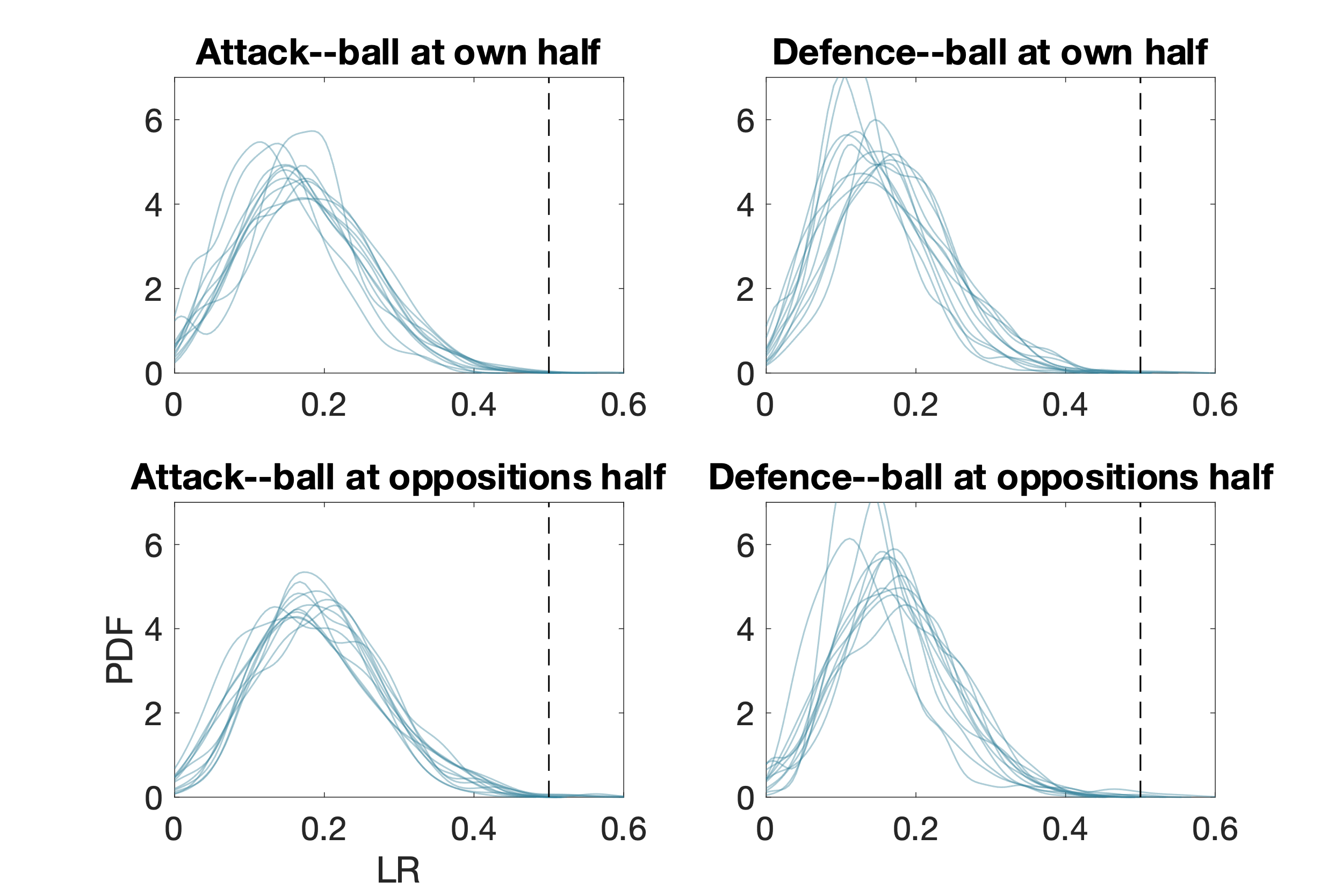


**S4: LR according to the ball position.** Distributions (probability distribution functions; obtained from kernel density estimates) of the ratio of the area of the inner convex hull over the area of the outer--each curve corresponds to data from specific events from one match. A few cases were discarded for having an insufficient amount of data (<500 data points). Dashed vertical lines show approximately where all the distributions end at 0.5.
